# The unbreakable journey: using photovoice to raise awareness and fight leprosy stigma in Papua, Indonesia

**DOI:** 10.1093/bjd/ljad059

**Published:** 2023-03-03

**Authors:** Ragil Dien, Hana Krismawati, Ivon Ayomi, Diana Timoria, Mary Chambers, Hardyanto Soebono, Marlous L Grijsen

**Affiliations:** Oxford University Clinical Research Unit Indonesia, Faculty of Medicine Universitas Indonesia, Jakarta, Indonesia; National Institute of Health Research and Development (NIHRD), Jayapura, Papua, Indonesia; National Institute of Health Research and Development (NIHRD), Jayapura, Papua, Indonesia; Oxford University Clinical Research Unit Indonesia, Faculty of Medicine Universitas Indonesia, Jakarta, Indonesia; Oxford University Clinical Research Unit, Ho Chi Minh City, Vietnam; Centre for Tropical Medicine and Global Health, Nuffield Department of Medicine, University of Oxford, Oxford, UK; Department of Dermatology and Venereology, Gadjah Mada University, Yogyakarta, Indonesia; Oxford University Clinical Research Unit Indonesia, Faculty of Medicine Universitas Indonesia, Jakarta, Indonesia; Centre for Tropical Medicine and Global Health, Nuffield Department of Medicine, University of Oxford, Oxford, UK

## Abstract

In preparation of an ongoing trial to improve the treatment of leprosy (MetLep, clinicaltrials.gov: NCT05243654), we conducted a photovoice project among persons affected by leprosy in eastern Indonesia. Photovoice is a participatory visual method in which photographic images are used to explore community health and social issues among disadvantaged populations. This project generated opportunities to visualize stigma and misunderstandings people with leprosy face, and the social and mental burden that this puts on them and those around them.

https://doi.org/10.1093/bjd/ljad059

Leprosy is a neglected tropical skin disease (skin-NTD) affecting the skin and peripheral nerves. Worldwide, reported new cases remain stable at around 200 000 per year.^1^ Although leprosy is curable, if insufficiently managed, it can result in permanent disabilities. An estimated 3–4 ­million people globally live with some form of disability because of leprosy, causing discomfort, disfigurement and loss in quality of life.^1^ People affected by leprosy, and their family members, are often heavily stigmatized, as myths and misconceptions surround the disease.^2^ The discrimination and physical disabilities faced by people with leprosy can limit opportunities to fully participate in education, employment and society.

Indonesia has the third highest number of people with leprosy in the world. Annually, over 17 000 new cases are identified nationwide, with high endemic pockets in eastern Indonesia. In preparation of a randomized trial evaluating adjunctive metformin to mitigate leprosy reactions (MetLep, clinicaltrials.gov: NCT05243654), we conducted a community engagement project to raise public awareness, promote critical dialogue, and reach policymakers about issues and concerns related to leprosy.

We used photovoice, which is a creative, participatory visual method, developed by Wang and Burris in the 1990s, to explore community health and social issues among disadvantaged populations.^3^ In photovoice, community members use photographic images that are taken and selected by themselves to capture aspects of their lives and environment, enabling them to represent and enhance their community, and giving them a voice to influence society and policymakers with the intention to provoke public debate and stimulate change.^4^ Photovoice has been implemented in vulnerable and minority populations around the world to deepen understandings of community concerns and to critically reflect on public health issues,^3^ for example refugees,^5^ people with mental or physical disabilities^6^ or those with chronic conditions, like HIV/AIDS.^7^

In January 2022, 30 persons with leprosy, their family members and healthcare workers volunteered to take pictures of their daily activities, everyday health and life conditions to document their concerns, struggles and strengths. They were guided through the process to provide narratives for the images through facilitated group discussions. Individual photographs were discussed for their meaning, contextualization and importance. Participants were encouraged to select three to five of their most compelling images to be exhibited during a photo exhibition in Jayapura, the capital of Papua province, to acknowledge World Leprosy Day (Figure 1). Over 250 people visited the exhibition, comprising community leaders, religious groups, nongovernmental organizations and local government officials. The images were compiled into a video that was broadcast at different venues throughout the city (https://www.youtube.com/watch?v=lYDTwt7vnRs). All participants provided written informed consent. Short interviews and feedback surveys were conducted among participants and visitors for evaluation.

This project generated opportunities to visualize the stigma and misunderstandings that people with leprosy face, and the social and mental burden this puts on them and those around them. Kristian, a family member, explained that his family abandoned their cousin, who was affected by leprosy. He regrets his past actions: ‘*If I could turn back time, I would not have let my cousin fight alone against leprosy*.’ Participants reflected that they felt ‘heard’ after sharing their photos and stories with others. Daniel, a person with leprosy, disclosed, ‘*My mother pushed me to join this project and meet others who are affected by leprosy. People were interested to hear my story. It gave me confidence.*’ A visitor attending the photo exhibition commented: ‘*Seeing the images and talking to the participants gives insight and meaning to their lives*.’

Photovoice is a powerful communication tool for health promotion and advocacy. A project among Maasai women in Tanzania to prevent and manage trachoma offers a great example of how the method helped to educate and empower the women to share their knowledge within their own communities.^8^ In Nigeria and Liberia, the methodology was used to help people affected by severe stigmatizing skin diseases, including leprosy, to identify challenges and design peer-led solutions to improve community health and wellbeing (https://www.sightsavers.org/from-the-field/2021/05/living-with-skin-ntds/). To our knowledge, this is the first peer-reviewed publication on the use of photovoice in people with a skin disease.

Photovoice can empower underprivileged populations, including people with stigmatizing skin diseases, such as skin-NTDs or psoriasis, to gain a voice and address the needs of their society. It encourages participants to engage with the wider public and promote social change. Our project, set in a Papuan community, has helped to express the lived experiences and perspectives of people affected by leprosy and their household members. The project allowed them to connect with the community and local policymakers through the dissemination of their photographs and narratives demonstrating the devastating impact leprosy has on their lives. Experiences from this project are also valuable to optimize the acceptance of the MetLep Trial in the same communities.

**Figure 1:**
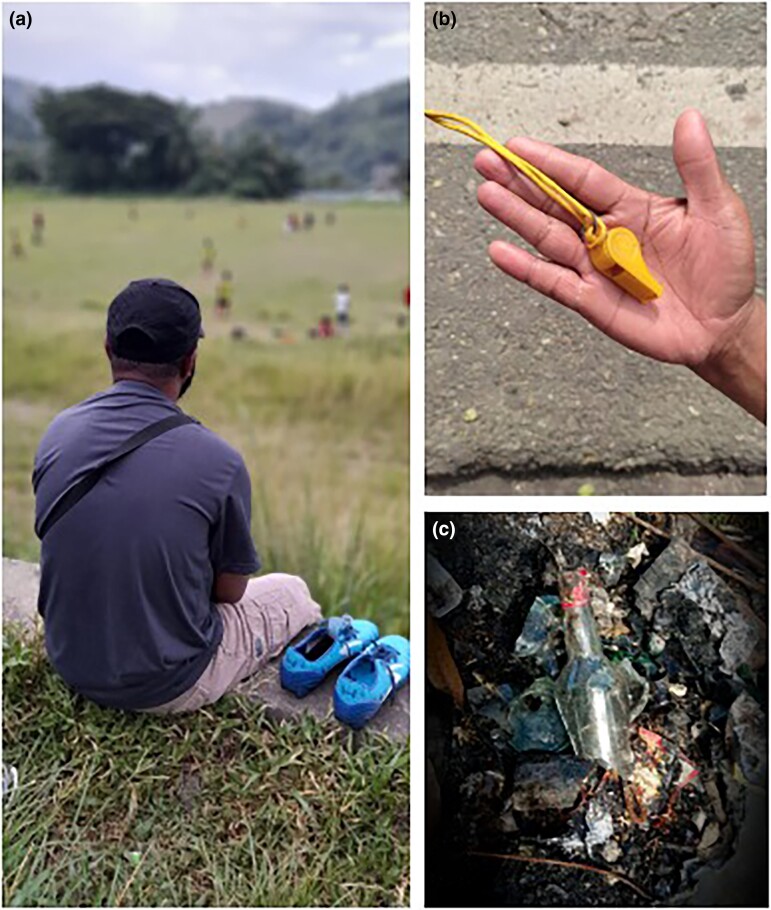
A compilation of three photographs plus captions that were presented by participants during the photo exhibition in Jayapura, Papua province, Indonesia, during World Leprosy Day 2022 [for privacy reasons, the original names of participants have been changed]. (a) Benjamin was fond of playing soccer since he was a child. He was selected to attend a football training abroad. Around the same time, he was diagnosed with leprosy. ‘*I was confused what to do. I decided to take care of my health and stopped playing soccer*.’ (b) Maria, a healthcare worker, was afraid of leprosy. ‘*One day when I parked my motorbike in front of the supermarket, a security officer approached me. I saw that some of his fingers were missing and knew it was leprosy. Back home, I washed my motorbike. I was afraid of getting the disease. I know better now. Leprosy is not transmitted this way and requires close contact for a long period of time.*’ (c) Johan, a person affected by leprosy: ‘*I was often invited to drink alcohol with my friends. In the beginning, I was able to refuse, as I was still on multi-drug therapy. But over time, I was influenced by the group and started drinking again. I lost the discipline to take my medication regularly. After some time, I motivated myself and stopped drinking to finish the treatment*.’

## Data Availability

Not applicable.
